# Translational neuroscience: the state of the nation (a PhD student perspective)

**DOI:** 10.1093/braincomms/fcaa038

**Published:** 2020-04-06

**Authors:** Caitlin Davies, Olivia K L Hamilton, Monique Hooley, Tuula E Ritakari, Anna J Stevenson, Emily N W Wheater

**Affiliations:** f1 Wellcome Trust 4-Year PhD in Translational Neuroscience, University of Edinburgh, Edinburgh, UK; f2 Centre for Discovery Brain Sciences, University of Edinburgh, Hugh Robson Building, 15 George Square, Edinburgh, EH8 9XD, UK; f3 Dementia Research Institute, University of Edinburgh, Chancellor’s Building, 49 Little France Crescent, Edinburgh, EH16 4SB, UK; f4 Centre for Clinical Brain Sciences, University of Edinburgh, Chancellor’s Building, 49 Little France Crescent, Edinburgh, EH16 4SB, UK; f5 Centre for Genomic and Experimental Medicine, Institute of Genetics and Molecular and Medicine, Crewe Road, Edinburgh, EH4 2XU, UK; f6 Centre for Reproductive Health, Queen’s Medical Research Institute, Little France Drive, Edinburgh, EH16 4TJ, UK

**Keywords:** translational neuroscience, research culture, industry partnership, public engagement, science policy

## Abstract

Many brain disorders are currently untreatable. It has been suggested that taking a ‘translational’ approach to neuroscientific research might change this. We discuss what ‘translational neuroscience’ is and argue for the need to expand the traditional translational model if we are to make further advances in treating brain disorders.

## Introduction

The World Health Organisation pinpoints brain disorders as ‘one of the greatest threats to public health’ (World Health Organisation, 2006), with one in four people affected by neurological or mental health conditions at some point in their lives (World Health Organisation, 2001). However, there is currently a dearth of treatments for these disorders. Translational neuroscience aims to resolve this by transforming knowledge gained from basic science into interventions and applications for treating human disease.

To understand why past decades of research have failed to result in successful treatments, we must understand what translational neuroscience is and how to effectively achieve it. Classically, translational neuroscience has taken the form of a ‘bench-to-bedside’ model, whereby laboratory research (‘bench’) directly informs the development of novel treatments or technologies in the clinic (‘bedside’). However, this model is reductive and neglects that translational neuroscientific research is cyclical, involving both wet and dry laboratory research, research culture, industry partners, the public and policy makers. In this article, we discuss the current landscape of translational neuroscience and the factors that influence its success in leading to novel treatments in the clinic.

**Figure:**
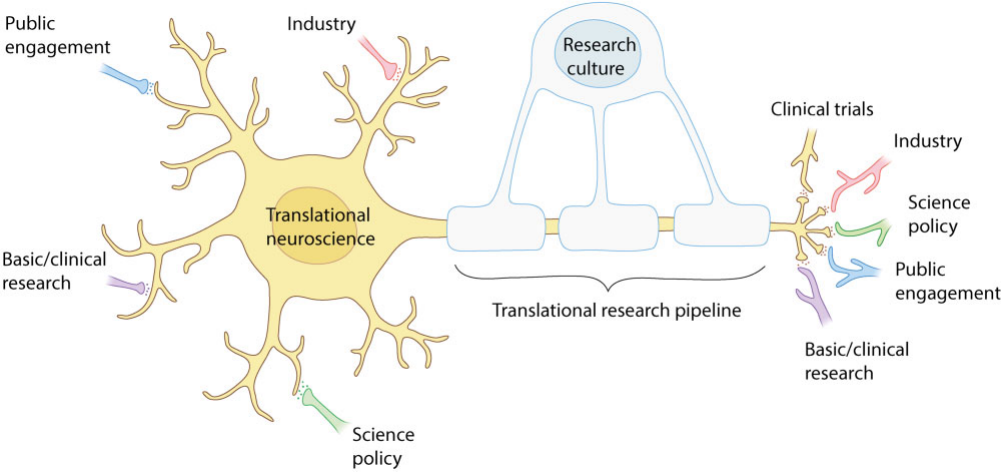


## Wet lab

At the core of translational neuroscience is the ‘wet’ laboratory research, which aims to understand underlying disease mechanisms and how they can be targeted for therapeutic purposes. One explanation for why this research has struggled to translate into the clinic may be the difficulty in studying the human brain in a laboratory setting. This is presently done using a medley of models from animals to human cells and post-mortem tissue; however, each is subject to inherent limitations in their availability, complexity and ability to recapitulate human brain function.

One of the most commonly used models for preclinical research is small rodents, mainly mice and rats. However, whilst animal models are indispensable for studying the intact, live nervous system, rodents lack the complexity of the human brain and do not fully recapitulate complex human disease. This is evident for instance in the field of Alzheimer’s research, where no single rodent model perfectly mimics all pathology seen in patients (Götz *et al.*, 2018). However, these models are continuously improving, and are useful for studying specific disease processes: for instance, transgenic mice and rats have been instrumental in understanding specific pathomechanisms of Alzheimer’s disease (Götz *et al.*, 2018), and were key in demonstrating the effectiveness of the antisense oligonucleotide therapy Spinraza before it moved onto clinical trials for spinal muscular atrophy (Corey, 2017).

A promising development for the field of translational neuroscience has been the advent of human induced pluripotent stem cell technology. This has been gaining popularity as a disease model, as it allows researchers to study dynamic disease processes in live human cells with practically unlimited material. This is in contrast to post-mortem studies or resection of live human brain tissue, both of which are highly informative of human brain function and disease, but much more limited in availability and modifiability. Furthermore, compared to rodents, human induced pluripotent stem cells provide a better tool for studying complex polygenic disease. However, human induced pluripotent stem cell-derived brain cells and organoids do not capture the complexity of the human brain, and similar to rodents are best used as a tool for studying specific disease processes.

In the absence of a ‘perfect’ model, the best approach is therefore to combine existing models in an attempt to increase the translatability of results. However, in order to best utilize the models currently available for translational research we need to understand the extent to which they can capture each aspect of disease. This not only requires a deep understanding of disease mechanisms seen in patients but also of the human brain and its functional diversity in general. This need for basic neuroscience research as a basis for translation is clear when considering the study of glial contributions to disease: while glia are increasingly recognized as contributing to neurological disease, glial research is an emerging field where many questions are only beginning to be answered. For instance, different subtypes of astrocytes and microglia may differentially contribute to disease; however, what these subtypes are, and how they differ functionally from each other, is largely unclear. Furthermore, there is evidence to suggest that human and rodent glia may have functional differences (Bedner *et al.*, 2020), but research into any such differences is limited, and the implications for using rodent glia for studying human disease remains unclear. This highlights how successful translation of research into the clinic will require input not only from translational neuroscientists but also from basic researchers aiming to characterize the complexity of the human brain.

The best approach we therefore have for studying human disease is to combine the available animal and human models to best recapitulate the disease process of interest, as well as to continue developing new models that may better capture the complexity of the human brain. However, it is important to bear in mind the limitations of both our current tools as well as our understanding of the human brain in trying to narrow the gap between preclinical research and clinical trial success.

## Dry lab

One area of translational neuroscience that is often lost in the typical bench-to-beside model, is that of ‘dry labs’. Dry labs typically utilize computational or statistical approaches to analyse data, encompassing both basic biology and disease pathology, as well as epidemiological approaches—addressing why, and to whom, neurological and psychiatric disorders occur.

In a translational context, examples of the type of output from dry labs include directly biological concepts, such as the analysis of ‘omics’ for target intervention and medical imaging for diagnostics. In addition to this, dry labs enact more overarching approaches such as text mining of health records, and large-scale systematic reviews and meta-analyses. The latter of these represents a route by which to amalgamate the outcomes of research and identify consensuses in the literature, which is crucial to then facilitate sound, evidence-based clinical decisions. The reconciliation, synthesis and appraisal of research is vital for effective translation in the era of the replication crisis, and when promising preclinical outcomes have failed to translate to positive results in clinical trials. By robustly analysing the literature, and systematically assessing why things have gone wrong, there is scope to both improve the design, conduct and analysis of neuroscientific research, as well as to pinpoint where future work should be targeted.

Another area of dry lab research that has greatly expanded in recent years, and offers a promising avenue for translational outcomes, is the analysis of data accrued through large-scale cohort studies and biobanks. DNA sequencing, brain scans, cognitive tests and output from wearable devices are just some of the data gathered by these studies, all of which can be analysed with the aim of delineating disease aetiologies or identifying biomarkers. Though typically somewhat biased, due to selection biases and participant attrition, these studies allow for health data to be scrutinized at a far larger scale than was previously possible. Additionally, while historically recruiting presenting patients, studies of specific diseases have become cognizant of the need to enrol at-risk participants in order to understand the therapeutic window for prophylactics. This is particularly important for conditions with long latencies or prodromal periods such as Alzheimer’s and Parkinson’s disease. Improved data linkage to NHS records within these studies is likely to provide more insight into longitudinal disease trajectories and allow for feedback from the clinic to be incorporated into research and analyses. These vast biomedical datasets ultimately need to be transformed into hypotheses and knowledge, which relies upon integrating the expertise of computer scientists and statisticians, with biologists and neuroscientists. Often there is too large a divide between these disciplines and leveraging the complementary skills of both, so tenets of one area are more readily translated to the other, is likely to expedite robust patient outcomes.

Each type of work that is conducted in a dry lab represents an incremental shift towards an outcome in the clinic, and can both feedback to the wet lab, and feedforward to clinical research. For example, identification of genes associated with diseases and outcomes in large-scale genome-wide association studies can inform model organism work, and also feed-forward into patient stratification or prognostication. The latter of these will likely be key in precision medicine and may lead to breakthroughs in therapeutics that have previously failed when targeted incorrectly. As with each area of translational medicine, neuroscientists in dry labs must keep in mind what is considered a meaningful outcome for the patient—be that feeling, function or survival—and ultimately target research in this manner.

## Research culture

Whether working in wet or dry lab settings, the working environment, or ‘culture’, of a research institution has the potential to promote or prevent the translation of research. One aspect of research culture under increasing scrutiny is the pressure to publish. With publications often seen as a form of academic currency, pressure to publish high quantities of research, quickly, and in high impact journals, is experienced by many researchers. As most high impact journals tend to favour novel or news-worthy findings, in its most extreme form, pressure to achieve impactful publications can lead to the misrepresentation of data. Examining 20 621 papers published in 40 scientific journals between 1995 and 2014, a 2016 study by Dr. Elisabeth Bik found that 3.8% of papers contained inappropriate image duplication, and in over half of these cases the type of image duplication was suggestive of intentional manipulation (Bik *et al.*, 2016). Whilst the prevalence of intentional misrepresentation of data in academic publishing is unclear, such cases exemplify how pressure to publish contributes to the crisis of scientific reproducibility. Pressure to publish novel or news-worthy results also de-incentivises replication studies that seek to confirm findings, and to validate both new and well-established methodologies—a key step in the translational process. The British Neuroscience Association have recognized that under these pressures, scientific knowledge is vulnerable to a bias that ‘skews scientific understanding, contributes to hyped expectations, and jeopardizes the translation of research to real-world applications’ (British Neuroscience Association, 2019).

Another way in which research culture can limit the translational potential of research is via its toll on the health and wellbeing of researchers. In January this year, Wellcome published the results of a survey that invited over 4000 researchers to share their views on research culture (Wellcome, 2020). Whilst many respondents viewed their research roles as a vocation, many highlighted the proliferation of metrics, multiple commitments, long working hours, lack of diversity and inclusion, short-term funding and lack of job security as prominent features of their research environments. Together with ‘publish or perish’ pressures, these factors see many committed researchers leave the sector, draining translational pipelines of skills and expertise.

Increasing awareness of these issues has prompted responses from organizations such as the Academy of Medical Sciences, Wellcome and the Medical Research Council, among others. The British Neuroscience Association, for example, have launched a Manifesto for Credibility in Neuroscience (British Neuroscience Association, 2019), aiming to encourage scientific rigour and support researchers in challenging damaging practices. A crucial feature of this manifesto is training in open science practices: a set of principles and tools that enable transparent, open and reproducible science (see The Centre for Open Science: www.cos.io, see also Wilkinson *et al.*, 2016). Pre-registration of analysis plans via the open science framework (www.osf.io) or publication of pre-registered reports, enable researchers to pre-define their methodologies, minimizing bias during the research process and, in the case of pre-registered reports, ensuring publication of results regardless of the outcome. Making data or code publicly available via secure repositories is another method of encouraging transparency in the research process, and also enables the validation of empirical work in external samples. Pooling expertise and making resources open and available fosters collaboration by bringing together researchers from different groups within the translational cycle. Platforms such as the open science framework facilitate collaborative project management, and cater for different institutional requirements by offering features such as embargos on public pre-registration documents until project completion, which may be required by industry partners. Upon completion of studies, manuscripts can be uploaded to pre-print servers such as biorXiv, medrXiv or the Wellcome Open Research platform (for those funded by Wellcome), which make manuscripts publicly available (with a citable digital object identifier). Pre-registration also prevents manuscripts from being held up in the publishing pipeline and opens them up to informal public review.

Funding bodies and research institutions can affect positive shifts in research culture from the top down by incentivizing rigorous scientific practices, committing to open science principles, valuing leadership skills and rewarding positive, supportive research environments in assessment frameworks for funding and career advancement. With this in place, from the bottom up, researchers will be encouraged to embed open principles in their work and utilize their expertise in the translational cycle.

## Industry

The translation of academic output into novel therapeutics relies on the investment of time and capital from ‘big pharma’. Pharmaceutical companies are licenced to research, develop, market and distribute drugs. Typically, the process of drug development takes 10–15 years, generally includes a combination of *in vitro* studies, *in vivo* studies and clinical trials, and can cost billions of pounds.

In recent years, venture capital funding has been pouring into biotechnology companies. This has facilitated a thriving biotech research and development culture and, in some cases, has allowed biotech companies to carry out early-phase clinical trials, increasing their value before acquisition by ‘big pharma’. This model of translating research reduces the risk for pharmaceutical companies and allows them to invest in a range of therapeutic strategies and disease areas. The spinal muscular atrophy drug, Spinraza, is an excellent example of how collaboration between academia, biotech and pharma can translate research into life-changing therapies. A deep understanding of the biology of spinal muscular atrophy was developed in academia, in particular at Cold Spring Harbor Laboratory and the University of Massachusetts. Subsequently, Cold Spring Harbor Laboratory and the biotech company Ionis began collaborating to develop the therapy. This led to a partnership between Ionis and the pharmaceutical company Biogen, and in 2015 Biogen acquired an exclusive licence for the drug. Following successful clinical trials, Spinraza was approved by the Food and Drug Administration and the European Medicines Agency as the first drug to treat spinal muscular atrophy.

Unfortunately, neuroscientific clinical trial success stories are few and far between. This has caused pharmaceutical giants such as Pfizer, Bristol-Myers Squibb, GlaxoSmithKline, AstraZeneca and Amgen to steer their pipelines away from neuroscience despite there being a huge unmet clinical need. Nevertheless, others such as Biogen, Takeda, Roche and Johnson & Johnson have maintained an important presence. The withdrawal of these major companies from neuroscience research and development has been a wake-up call for the community. To incentivize future investments, lessons must be learned from previous failed trials. A prevalent theory as to why trials are failing is that there is a lack of understanding of the mechanisms underlying neurological and psychiatric disorders. Perhaps a stronger emphasis on collaboration between industry and academia would ensure that the capital and project development expertise of ‘big pharma’ is coupled with deep biological expertise in academia, and therefore accelerate the understanding of neurological diseases and mental health conditions. Another issue may be that outcomes in clinical trials could be targeted and measured more effectively. To address this, companies such as Roche and Takeda are developing research programmes to collect digital biomarkers, using smartphones and wearable technology, from patients and clinical trial participants. These will provide a more comprehensive and perhaps more sensitive measurement of disease and patient response to treatment.

An often overlooked contribution of industry to the translation of research into therapeutics is in providing researchers with the tools to answer their fundamental questions. This flywheel of moving research into industry and injecting innovative technologies back into academia will be essential in driving impactful, robust neuroscientific research. Once the ground truths of neurological diseases are better understood, and clinical outcomes are more effectively measured, it will be a matter of time before pharma substantially reinvest to fulfil the unmet need of therapeutics for neurological and psychiatric disorders.

## Public engagement

Public engagement is another facet of research and training through which the translational gap can be narrowed. The National Co-ordinating Centre for Public Engagement defines public engagement as: ‘the myriad of ways in which the activity and benefits of higher education and research can be shared with the public’ (National Coordinating Centre for Public Engagement, 2018). This definition encompasses activities such as science communication, a largely unidirectional interaction between researchers and the public, and also bi-directional models such as patient and public involvement (PPI) and priority setting partnerships.

The words ‘public engagement’ may conjure a pedagogical model whereby researchers disseminate information to the public through a range of activities: stands at science festivals, public talks and engaging with the media. This is regarded as a responsibility of academia, and it is also a way of effectively communicating public health messages that are more specific to the brain. There is plenty of public awareness that smoking damages the lungs and that eating your ‘5-a-day’ is beneficial for cardiovascular and metabolic health. But both of these are also true for the brain. The links between brain health and these lifestyle factors tend to get left out of public health messages, a gap which public engagement could fill.

PPI strategies are common in clinical trials and have great potential to improve translation. PPI includes involving patients in developing trial ideas or planning logistics, which can better take into account participants’ needs. This, in turn, improves enrolment to trials and, possibly, participant retention in long-term studies (Crocker *et al.*, 2018). PPI is now also being adopted in basic science settings. A recent example of this, from the University of Edinburgh, is the ‘Buddy Pairs’ scheme. Here, dementia research laboratories were paired with individuals who had lived experience of dementia (both patients and carers; Kennedy *et al.*, 2019). The scheme involved lab tours to facilitate knowledge exchange within these pairs. Visitors with dementia may be curious to see what a brain looks like with their disease, or about how their medication works in the brain. By engaging with patients, researchers learn that maintaining a higher level of independence, or delaying disease progression, are often more important to patients than the development of treatments or cures. Interacting with patients may also provide a powerful motivation for researchers to continue working in research. As in the clinical trial setting, PPI is seen as an important part of the strategy to encourage people to participate in research studies and to be informed about what their participation enables.

Priority setting partnerships go a step further by producing a tangible result from the exchange between stakeholders. The James Lind Alliance facilitates priority setting partnerships through a formalized process in which patients, carers and clinicians, are consulted. This process involves surveys and workshops in order to converge on the stakeholders’ top 10 priorities. These may not necessarily be specific research questions but rather important areas for future research. Much like PPI, the ‘Top 10s’ often challenge preconceptions held by the research community about what is important to patients. While much research effort is spent on developing a cure, priorities set in the priority setting partnership may focus on improving quality of life through earlier diagnosis or better management of distressing symptoms. As an evidence base in and of themselves, the top 10 priorities are also a useful resource for justifying funding of research areas that might otherwise go neglected. For example, since the Parkinson’s disease priority setting partnership, published in 2014, the James Lind Alliance reports three studies into anxiety and depression management in Parkinson’s disease in response to the priority item: ‘What approaches are helpful for reducing stress and anxiety in people with Parkinson’s’ (James Lind Alliance, 2014).

Public engagement has the capacity to improve translation in a number of ways. Dissemination-as-intervention, with regards to the impact of lifestyle factors on brain health, is an important supplement to public health messages that do not usually focus on the brain. Additionally, by positioning patient groups and carers as partners in research, public engagement contributes to the success of trials, reveals novel research opportunities and fosters positive feeling towards the research community.

## Science policy

An often-neglected arm of the translational neuroscience model is that of science policy, a term with a somewhat broad definition. Principally, its goal is to consider how science and technology can best serve the public, and form and enact public policies accordingly. Whether by directly implementing research findings into policy-making decisions, influencing how certain fields are funded, or by supporting the translation of emerging technologies, changes to science policy are powerful means through which research can shape reality and improve the health of individuals and populations.

The relationship between research and science policy is bi-directional; basic and clinical research can inform public policies, and research itself can be impacted by policy. The former is illustrated by the development of specialized stroke units in UK hospitals, widely considered a landmark innovation in stroke care. Annually, more than 100 000 people in the UK experience a stroke. Research has shown that provision of care in a specialist stroke unit, versus a general medical ward, reduces rates of mortality and disability, and enables patients to retain greater independence post-stroke. This directly translated into policy, with the Department of Health recommending major changes to the stroke care system in their 2007 National Stroke Strategy, listing dedicated stroke units as ‘the single biggest factor that can improve a person’s outcomes following a stroke’ (Department of Health, 2007). Nonetheless, ensuing research suggests there remains room for improvement, and sustained engagement with stakeholders and policy makers is vital for further advances in neurological disorder treatment and care.

Science policy also impacts research, most notably through determining allocation of public money for research funding. The more nuanced effects of policy are embodied by the complex issues surrounding mental health research and psychoactive substances. In recent years, novel psychiatric drug development has slowed and access to psychological interventions is sub-optimal. Research involving illegal drugs, such as LSD, ketamine, psilocybin and MDMA, points to their potential as treatment strategies for various mental illnesses (Schenberg, 2018). Although the use of these substances in research is legal, they remain strictly regulated. Such policies, unfortunately, make working with controlled substances more challenging. This stymies investigation of clinical efficacy and mechanistic understanding of their activity. What is more, the stigma that surrounds their usage potentially discourages patient engagement and dissuades involvement of researchers, institutions and funders. Policies that hinder research should be discussed, debated and challenged, and ultimately the scientific evidence should be allowed to prevail.

The above examples outline how science policy can both facilitate and frustrate the translation of research into meaningful healthcare solutions for the public. Researchers and their institutions are ideally positioned to influence public policy and foster better translation. They possess the expertise crucial for promoting evidence-based policy making and are largely perceived as knowledgeable, trustworthy and without vested interests—traits that are sometimes few and far between in political spheres. Despite this, research findings do not always form the basis of health-related policies, a phenomenon dubbed the ‘research/evidence-policy gap’. A wide variety of factors contribute to this discordance. Policy makers are seldom scientists and are required to make decisions over short timescales, limiting the amount of information they can amass and interpret before reaching a decision. Scientists rarely understand the intricacies of policy making or how to communicate core information in a manner that can be readily used by decision makers. Additionally, existing academic structures do not incentivize researchers to participate in policy-related processes.

To address the ever-increasing public health and economic burden that neurological and psychiatric disorders present, research must be translated into effective science policy and researchers should play an active role in this. As manifested by the success of specialized stroke units, closer alignment of evidence and political decision-making has huge potential to improve patient outcomes. This will not occur without better engagement between all involved parties and is an area that remains a translation gap for policy makers and scientists alike.

## In summary

The translation of fundamental neuroscientific research into meaningful clinical outcomes requires an expansion of the traditional ‘bench-to-bedside model’. The translational cycle includes academic researchers (from both wet and dry lab settings), industry partners, individuals with lived experience of target diseases or disorders, and policy makers. We have highlighted the necessity of collaboration between these groups, who together can identify appropriate clinical questions, select suitable methodological approaches and validate them, replicate findings, develop tools and technologies that drive discovery, and translate these discoveries into treatments or policy changes that aim to improve public health. Interactions between these groups are bi-directional and confer mutual benefits. The role of research institutions lies at the centre of this translational cycle. Through the establishment and maintenance of healthy research cultures, which incentivize scientific rigour, and support engagement with industry partners, community stakeholders and policy makers, researchers can more effectively lay the scientific groundwork required to deliver meaningful clinical outcomes for those who need them.

## Funding

C.D., M.H., T.R., A.J.S. and E.N.W.W. are supported by funding from the Wellcome Trust [203972/Z/16/A to C.D.; 203801/Z/16/Z to M.H.; 203758/Z/16/Z to T.R.; 203771/Z/16/Z to A.J.S.; and 203769/Z/16/A to E.N.W.W.]. O.K.L.H. is supported by funding from the College of Medicine and Veterinary Medicine, University of Edinburgh. All authors are enrolled on the 4-year PhD in Translational Neuroscience - training the next generation of basic neuroscientists to embrace clinical research at the University of Edinburgh.

## Competing interests

The authors report no competing interests.

## Abbreviations

PPI =: patient and public involvement
